# Introducing Low-Fidelity Simulation Teaching in the Early Years of Undergraduate Medical Training

**DOI:** 10.7759/cureus.89521

**Published:** 2025-08-06

**Authors:** Joshua Garg, Thomas McLelland

**Affiliations:** 1 Medical Education and Simulation, Queen Elizabeth Hospital Birmingham, Birmingham, GBR; 2 Medical Education and Simulation, Sandwell and West Birmingham Hospitals NHS Trust, Birmingham, GBR

**Keywords:** simulation in medical education, teaching clinical skills, undergraduate clinical skills education, undergraduate medical student, undergraduate teaching strategies

## Abstract

Introduction: Simulation is a widely used educational tool for both undergraduate and postgraduate medical education. There are different levels of simulation fidelity; low fidelity uses simplistic models but is often cost/time effective, whereas high fidelity tends to bring more realism but is resource-intensive. In undergraduate medical training, simulation is often used for students in their final year of medical school prior to qualifying as a doctor, and therefore, the literature is limited with regard to students in their earlier years of training. The aim of this project was to create cost/resource-effective, low-fidelity simulation training sessions for more junior medical students to help them consolidate skills learnt in isolation, in turn facilitating higher levels of critical thinking.

Methods: We developed low-fidelity simulation sessions for third-year (first year clinical) medical students. These comprised clinical scenarios that would encourage students to apply skills that were previously taught or assessed in isolation, to form a holistic clinical assessment of a patient. Students were given a pre- and post-session questionnaire to help ascertain the usefulness of the sessions.

Results: Thirty-two students took part in the simulation sessions. Questionnaires were used to ascertain their level of confidence in the full assessment of a patient. Their pre- and post-session feedback was compared, and the subjective scores were 4.09/10 and 8.25/10, respectively (p<0.05). Qualitative feedback suggested that the students enjoyed the clinically adjacent nature of the scenarios and felt that they were able to consolidate the knowledge they had obtained in pre-clinical years.

Discussion/conclusions: We demonstrated that students in earlier years of training can benefit from simulation teaching. Running the sessions was light on resources and an effective tool for students to consolidate skills and apply higher levels of thinking, preparing them for future years of training.

## Introduction

Simulation training has become an integral part of both undergraduate and postgraduate medical education. It is a particularly useful tool in providing undergraduate medical students with the opportunity to apply knowledge and skills, learnt in isolation, to real-life scenarios of patient management [1]. The aim of simulation training is to bridge the gap between theoretical knowledge and practical medicine, improving ease and confidence in the latter [2]. The efficacy of simulation as an educational tool is based upon experiential learning theory, which describes the learning process as situational and environment-based [3]. Simulation training aims to replicate perceived stress and distraction experienced in the clinical environment, with the primary focus on human factors and increasingly complex decision making [4].

Simulation training has developed significantly over recent years with increased sophistication of the interactive mannequins utilised [5]. The use of models with higher levels of interactivity and realism separates low and high-fidelity simulation methodology. There is a school of thought that for successful implementation of simulation into medical training, participants must suspend disbelief, hence the drive to increase realism [6]. It is unsurprising, therefore, that the intuition from medical educators is that high-fidelity simulation is superior to low-fidelity equivalents. There is, however, growing evidence that there is no inferiority of educational outcomes when comparing low to high-fidelity simulation [1,7], and involvement in high-fidelity simulation training can lead to an inflated self-confidence, which may translate into troublesome risk-taking [8]. This is an important consideration when designing educational tools, particularly in the early years of undergraduate medical education, when the educational aim should be to develop foundational skills.

Much of the data supporting the use of simulation training in undergraduate medical studies is from research focusing on medical students in senior years and the use of high-fidelity models. Repeatedly, one of the key aspects of negative feedback expressed by medical students, at completion of study, is a lack of readiness for clinical practice [9]. One avenue to address this deficiency is through early exposure to clinical scenarios designed to combine skills and cultivate higher thinking.

Key educational domains that simulation training aims to develop include teamwork, resource management, and skill acquisition, all of which are equally as effectively addressed with low compared to high-fidelity simulation models [10]. High-fidelity simulation is accompanied by a significant time and financial burden, from mannequin procurement to educator training on the use of required hardware and software. Therefore, if an alternative educational method is available with equivocal knowledge enhancement and improved confidence in managing patients, then this must be seen as a positive in the early years of study.

Herein, we discuss our development of a novel low-fidelity simulation series aimed at introducing students in pre- and early clinical years to the concepts of patient communication, examination, investigation, and management planning. During these earlier years of study, students are often doing aspects of a patient assessment in isolation, e.g., a history or examination in the form of workplace-based assessments (WPBAs). We aim to demonstrate the efficacy of using simulation as an educational tool at an early stage in an individual’s medical career, introducing the basic structure and underpinning themes that may stand them in good stead for future clinical practice and educational activity engagement. Therefore, this study specifically aims to (i) assess the impact of low-fidelity simulation on student confidence in holistic clinical assessment and (ii) evaluate the feasibility and educational value of running these sessions in a resource-light format.

## Materials and methods

Study design**

The low-fidelity simulation sessions were designed for third-year medical students from a single medical school in their first clinical year (i.e., based in a hospital). They were selected from three different firms that were under the supervision over the course of their placement by two different clinical teaching fellows. Each student would take part in two separate sessions in small groups of 5-6 peers. Each session contained six different scenarios, meaning students would have the opportunity to lead at least one scenario in each session. The sessions would take place in a seminar room with the only equipment being a bed, low-fidelity mannequin, stethoscope, and printed or digital copies of the relevant investigation results (e.g., blood results, x-ray, etc.). Each scenario would be structured in a way that would start off with little information, with the objective being to take a focussed history, specific examination, and then think about initial investigations, which could then be interpreted to help them formulate appropriate differentials. The scenarios were picked based on the student's curriculum of core conditions that they are expected to be familiar with by the end of their third year (example cardiology case outlined in Table 2 in the Appendices). The session would be run by 1-2 clinical teaching fellows (post-foundation training doctors) who would act as the ‘voice’ of the patient and could simulate basic examination findings. Following each scenario, there would be adequate time (approximately 20-30 minutes) to debrief and discuss the case. The cases would be aligned with the students’ curriculum and based on medical knowledge they will have been taught during their pre-clinical years. There would be a range of scenarios from different specialties, including cardiology, respiratory, gastroenterology, and general surgery.

Data collection

Before the sessions, students were anonymously asked, subjectively, how confident they felt in the full assessment of a patient on a scale of 1-10 (1=not at all, 10=extremely confident). Upon completion of the sessions, the students were asked to complete an anonymous, subjective questionnaire (Table 3 in the Appendices) to help analyse the effectiveness of the teaching, as well as gain positive and constructive feedback on the teaching style and overall structure of the sessions.

Statistical analysis

A paired t-test was performed on the pre- and post-session scores to define statistical significance. A p-value of <0.05 was considered statistically significant. 

## Results

**Figure 1:**
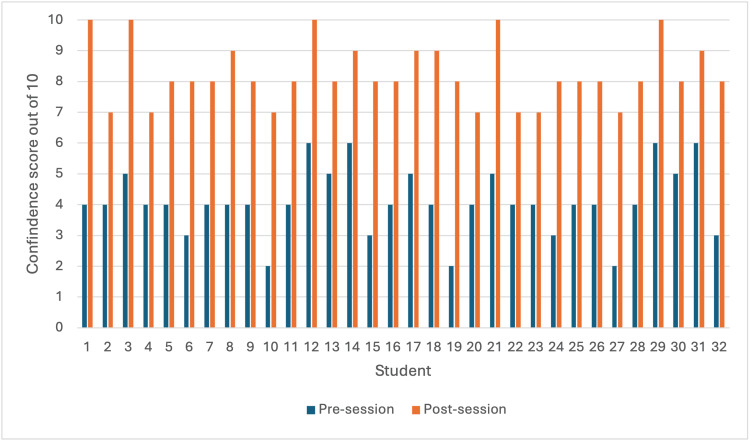
Students' pre- and post-session scores. Bar chart to show individual student’s pre- and post-session scores out of 10 when asked how confident they felt in performing a full assessment of a patient.

A total of 32 third-year medical students took part in the sessions and filled out their pre- and post-session questionnaires. Figure 1 shows the pre- and post-session scores out of 10 when asked “how confident do you feel with fully assessing a patient?”, with an average of 4.09/10 and 8.25/10, respectively (p = 2.34e^-20^). The range was 2-6/10 pre-session and 7-10/10 post-session. There was an average increase in the subjective score of 4.16 following the sessions. 

**Figure 2:**
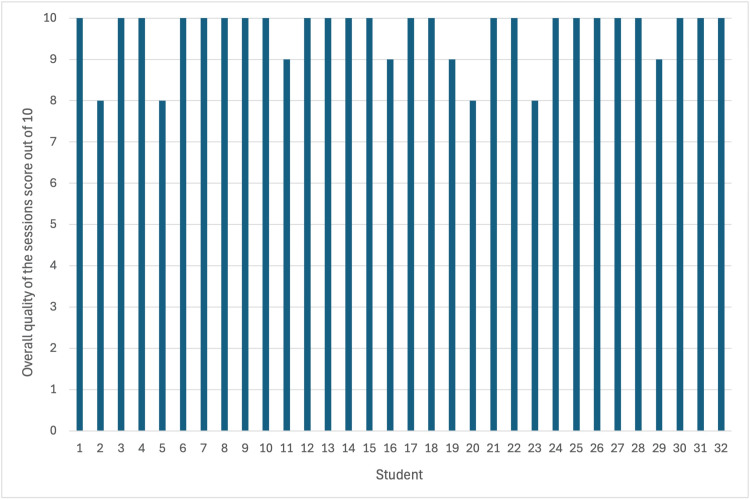
Subjective student scores for overall quality of the sessions. Bar chart to show scores out of 10 when students were asked how they would rate the overall quality of the teaching sessions with a mean score of 9.62/10.

When asked for feedback on the quality of the teaching, the average score of the overall quality of the sessions was 9.62/10 with 0 being very poor and 10 being excellent (Figure 2). The range of scores was 8-10. Although this is a subjective measure, it demonstrates that not only did the students enjoy the teaching, but the delivery was of good quality.

Anonymous, qualitative feedback showed that students enjoyed the hands-on approach of the low-fidelity simulation and felt that they were able to consolidate their prior knowledge (Table 1). They also enjoyed the initial vagueness of the scenario, allowing them to use their history and examination skills combined with interpretation of investigations to come to the most likely differential diagnosis. The feedback was overwhelmingly positive, with the only constructive point being to provide more sessions in this format.

**Table 1:** Qualitative feedback. Anonymous student feedback on the low-fidelity simulation teaching sessions.

Student	Response when asked: Is there anything that you particularly liked about the sessions?	Response when asked: Is there anything you would change or suggest for improvement?
1	Interactive, good energy and high level of details	Nothing
2	Interactive	No
3	The cases and explanation were great	N/a
4	Really useful for consolidating everything we’ve done and applying it to cases. At the correct level for our learning.	Nothing!
5	Liked the more vague cases	n/a
6	Hands on	N/a
7	Really helpful to have example cases that were quite vague in presentation to be able to do a motif of examinations and histories	-
8	The case scenarios that require both history taking and examination were very useful.	Nothing that I can think of. I found it very useful. The session was great!
9	Objective Structured Clinical Examination (OSCE) practice	N/A
10	Going through an entire case from history to management	Nothing, it was all useful
11	Being able to do history, examination, and interpretation in one	-
12	Interactive	No
13	I really liked the OSCE-style true cases	No
14	Interactive cases, from history to interpretation of tests, with opportunity to discuss with peers and come up with potential management options.	None, it was a really useful session, got a lot out of it
15	Interesting case examples. Good to put everything together	not sure
16	Going through the whole process of investigations, management, etc	N/A
17	The interaction and it covering all aspects of history and examination	N/A
18	Able to put everything I've learnt together	More teaching like this
19	Fun and interactive	-
20	I like that they made me think outside the box	No
21	Really useful and made me feel more prepared for exams and future years	N/a
22	More realistic cases with vague presenting complaints	Nothing, thank you
23	Being able to do different parts of an assessment together rather than relying on one part	-
24	Felt like I was doing a real patient assessment but in a safe environment.	Nil
25	Fully supportive sessions and interesting cases	No
26	It was all great, thank you	More sessions like this
27	Given independence and could think through an assessment instead of just listing off questions	-
28	Everything was really good	n/a
29	Felt encouraged to push myself in a productive way	More cases
30	Good chance to debrief after each scenario	-
31	Really fun way to learn and prepare for the future	No thank you
32	Getting to do the whole assessment	No

All of the students felt that the sessions improved their knowledge and skills, and therefore, their ability to complete a full clinical assessment and this was also statistically significant. When discussing student experience, compared to the usual assessment technique of limited patient assessment in the form of WPBA, students commented on the superiority of the use of simulation as an educational tool. They reported an added realism and degree of stress whilst participating in the scenarios, which participants found useful to dispel disbelief. Additionally, they appreciated the multitude of internal and external factors which make patient assessment a dynamic professional experience.

Feedback from those delivering the sessions showed that it was not only enjoyable to teach, but also straightforward to run. Faculty had only a short briefing before commencement of the sessions, with no prior hardware knowledge required, which provided them with flexibility to tailor the sessions to individual needs. It was also noted that the students generally performed well and that the concept of joining together different aspects of a patient assessment was not beyond their capabilities, despite the targeted early stage of training. 

## Discussion

Through the low-fidelity simulation sessions created, we have demonstrated that this form of teaching does not necessarily need to be reserved for more senior medical students, as is the focus of the existing evidence base. The lower fidelity nature is a cost-effective and time-conscious way of allowing medical students to utilise history, examination and investigation interpretation skills, previously learnt in isolation, to holistically assess a patient in a safe environment and promote higher levels of thinking. Not only did the simulation sessions demonstrate improvement in the students’ confidence, but they were also enjoyable to partake in as an educator. Relatively few resources are required, and the creation of the cases can be tailored easily to individual needs or curricula. 

Low-fidelity simulation does not fully recreate the more realistic and human factor-focused aspects that high-fidelity simulation does. Nicolaides et al. compared the performance of final year medical students who participated in both low- and high-fidelity simulation sessions [11]. They found that overall performance was higher in low-fidelity sessions, whilst reported stress levels were higher with high-fidelity models, as was the overall rating of the educational session quality. They concluded that high-fidelity was superior in improving leadership skills; however, low-fidelity models demonstrated significant improvement in technical/clinical skills. Whilst students are more junior, technical skills are the foundation for developing clinical leadership and management. Therefore, it can be argued that for third-year students to get the most out of these educational activities, lower-cost, low-fidelity teaching models are optimal. 

There was a consensus from the students in our study that doing aspects of an assessment in isolation, although initially important in the early years of training, feels unnatural when seeing real-life patients and unrealistic in clinical practice. There is a heavy focus on WPBAs (e.g., Mini-cex) whereby a student is observed conducting a history or examination in isolation. Although a useful tool to ensure basic skills are at the expected level, it can be difficult to get to the diagnosis without using other clinical skills. Our qualitative results show that students found the study sessions useful and helped to improve confidence in combining skills and knowledge obtained during their education to date. 

With comparisons to current literature, a study by Yu et al. also found that final year medical students had a statistically significant increase in their confidence levels after participating in high-fidelity simulation teaching sessions [12]. Whilst this is in keeping with the findings of our study, they used high-fidelity simulation, and this was conducted on more senior medical students than the target audience of our educational series. There is limited published research for simulation teaching in early clinical year students. A study by Moll-Khosrawi et al. reported that participation in high-fidelity simulation-based education did not enhance or stimulate the motivation of third-year medical students when compared to seminar/lecture-based teaching [13]. These results differ from this study, as students reported that they enjoyed this format of teaching and requested increased frequency. Additionally, with regard to clinically relevant learning, students commented on their preference for simulation training over the individual components assessed during WPBAs.

Another piece of primary research by Philp et al. focused on the use of low-fidelity simulation, using simple resuscitation hardware and peer role-play, to deliver specific cardiology teaching [14]. Fourth-year medical students, as the targeted demographic in this study, reported improvement in confidence across nine relevant clinical skills, many of which were incorporated into our simulated scenario designs. They also commented on the preferential use of low-fidelity simulation to ensure the educational safety of the participants. High-fidelity models are associated with higher biological and subjective stress measures [15], an important consideration when designing educational events at an early stage of study, where unsatisfactory educational experiences can have a significant impact on future engagement. 

A limitation of our study is that we have no objective marker to determine the effectiveness of the sessions with regard to formal exam performance. Our analysis is purely subjective; however, the sessions were designed more as an educational tool to promote more realistic patient assessment as opposed to passing exams. In a similar way to informal progression assessment through WPBAs, clinical skills are formally examined in isolation during the early years of medical school. 

Another limitation of our study was that we did not have a control group to compare the educational outcomes with. We considered using a more traditional lecture style teaching method to act as a control, however felt that this would disadvantage those students who were not having first-hand involvement with simulation training, given the available positive educational data. We also wanted to provide an educational series, with repetition and broad clinical exposure, and therefore, we did not have the resources to control across these sessions. Additional limitations include a relatively small sample size and students being from a single institution. 

## Conclusions

In conclusion, we feel that utilisation of low-fidelity simulation in the early clinical years of medical training is a useful and enjoyable educational tool that can be applied in a variety of educational settings. It allows students to build confidence and promote more critical thinking when assessing patients and is conducive to a holistic approach. Students in their junior years often learn and get assessed on clinical skills in isolation; however, pushing students further at an earlier stage can not only enhance their learning but also builds a good foundation for their ongoing training. The low-fidelity style is conducive to this as it provides a safe environment with minimal stress and can therefore nurture optimal performance and dedicated learning. We envisage simulation training as a tool to accumulate knowledge and build on previous performance throughout clinical years, with repetitive use improving familiarity and comfortability in these scenarios. 

The key takeaways from this project are that low-fidelity simulation sessions are adaptable, easy to create, low-cost and can be delivered with relatively few faculty members to a wide range of audiences, with educational value gleaned to students of all abilities. Further research is needed with these specific student demographics, alongside objective ways of measuring outcomes. 

## Appendices

**Table 2:** Example scenario template. Example cardiology scenario for acute coronary syndrome. Credits: Joshua Garg and Thomas McLelland.

Sub-section of scenario	Details
Scenario Title	Acute coronary syndrome
Initial information	You are in A&E and asked to see a patient with chest pain. Please take a focused history and appropriate examination.
History points	52-year-old male. Sudden onset chest tightness lasting for the past four hours. Associated shortness of breath (SOB) and sweating. Pain is mainly left side of the chest. Usually fit and well, has been more SOB lately on exertion. 20 pack-year smoking history, no significant family history (FH).
Examination findings	News = 1, HR 99, RR 20, Sats 98% RA, BP 165/99, Temp 37.6. Appears clammy. Pulse regular, normal heart sounds, and chest clear. No other significant examination findings.
Prompt for differentials	ACS, PE, pneumothorax, and MSK
Suggested management	ECG, CXR, analgesia, Bloods (FBC, U+Es, Trop-T, consider D-Dimer based on Well's score), and senior review
CXR	Normal
ECG	Sinus rhythm. ST elevation V2-4
Bloods	Hb 134, Plt 243, Wcc 6.7, U+Es normal, and Trop-T 433
Working diagnosis	Acute STEMI
Further management	Alert cardiology and ACS management
Equipment provided	Low-fidelity mannequin, voice of patient provided by faculty, stethoscope, ECG, blood results, and CXR

**Table 3:** Student questionnaire. Questionnaire given to students to assess the quality and effectiveness of the simulation sessions. Credits: Joshua Garg and Thomas McLelland.

Question number	Question	Answer
1	Before the sessions, how confident did you feel in the full assessment of a patient on a scale of 1-10? (1=Not at all, 10=Extremely confident)	
2	Following the sessions, how confident do you feel in the full assessment of a patient on a scale of 1-10? (1=Not at all, 10=Extremely confident)	
3	Is there anything you particularly liked about the sessions?	
4	Is there anything you would change or suggest for improvement?	
5	Overall, how would you rate the quality/usefullness of the sessions? (1=Poor/not useful, 10=Excellent/very useful	
